# Endophytic Fungal Diversity and Its Interaction Mechanism with Medicinal Plants

**DOI:** 10.3390/molecules30051028

**Published:** 2025-02-24

**Authors:** Yuan Gao, Yan Xu, Zhijia Dong, Yuyang Guo, Jianghan Luo, Fuling Wang, Lijun Yan, Xiang Zou

**Affiliations:** 1 School of Pharmacy, Harbin University of Commerce, Harbin 150076, China; 18252583359@163.com (Y.X.); tyharuka0930@163.com (Z.D.); 18846086109@163.com (Y.G.); 102690@hrbcu.edu.cn (J.L.); iwfl86@163.com (F.W.); ylj@hrbcu.edu.cn (L.Y.); 2 Engineering Research Center of Natural Antineoplastic Drugs, Ministry of Education, Harbin University of Commerce, Harbin 150076, China

**Keywords:** endophytic fungi, medicinal plants, secondary metabolites, interactions, diversity

## Abstract

This paper reviewed the diversity of endophytic fungi and their interactions with medicinal plants, along with the research methodologies utilized to investigate these interactions. It mainly includes the diversity of endophytic fungi, as well as distribution diversity, species diversity, and the diversity of their metabolites and functions, including antibacterial, anti-inflammatory, anti-tumor, insecticidal, antioxidant capabilities, and so on. The research methodologies employed to investigate the interactions between endophytic fungi and medicinal plants are categorized into metagenomics, transcriptomics, metatranscriptomics, proteomics, and metabolomics. Furthermore, this study anticipates the potential applications of secondary metabolites derived from endophytic fungi in both medicine and agriculture.

## 1. Introduction

Endophytic fungi reside within the tissues and organs of their host plants without evidently harmful symptoms for all or part of their life cycle. Endophytic fungi, as opposed to parasites or diseases, are able to establish mutualistic partnerships with plants in which both sides gain from the equilibrium. Plants are able to create compounds like iron transporters and plant hormones that improve their resilience to stress because of endophytic fungi.

The secondary metabolites produced by endophytic plants play a crucial role in protecting cells by scavenging harmful free radicals [1]. Concurrently, endophytic fungal secondary metabolites have emerged as a valuable source for the creation of pharmaceuticals with anti-inflammatory, anti-tumor, anti-cancer, and antioxidant characteristics [2]. Endophytic fungi also have demonstrated a great deal of promise for advancement in the field of agriculture in recent years. It is anticipated that some endophytic fungi with insect-resistant qualities may develop into novel pesticides. Furthermore, a great deal of research has demonstrated that the application of endophytic fungi that are capable of fixing nitrogen and solubilizing phosphorus in combination with traditional agricultural fertilizers can greatly enhance plant growth indicators [3,4,5,6]. Therefore, in order to promote the application of endophytic fungi in the field of medicine and biological control, it is necessary to study the interaction mechanism between endophytic fungi and plants.

**Figure 1:**
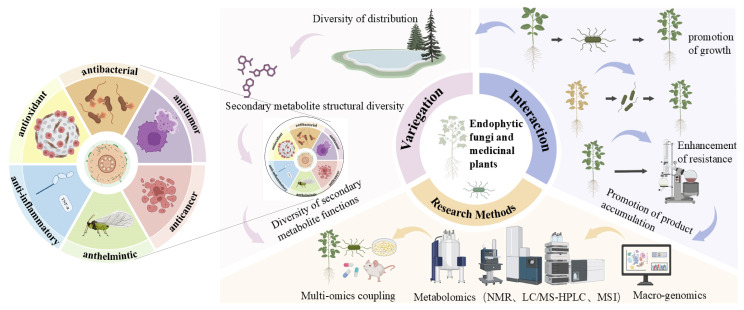
Endophytic fungal diversity and its interaction mechanism with medicinal plants. (Adapted from [7,8,9,10,11,12]).

Multi-omics-integrated technologies are currently being progressively applied to the study of endophytic fungal diversity and the mechanisms of interaction between endophytic fungi and medicinal plants, building on the foundation of classic omics technologies. By utilizing SCIENCE DIRECT, WEB OF SCIENCE, and PUBMED, search for literature from the past five years in 2024 using keywords such as “endophytic fungi”, “medicinal plants”, “interactions”, “growth promotion”, “increased antagonism”, “secondary metabolites”, and “omics”, and further screen the literature for use in this article. This review mainly discusses the diversity of endophytic fungi and the interaction mechanism between endophytic fungi and host plants from the above aspects and the application of omics technology in the study of the interaction mechanism between endophytic fungi and medicinal plants, as shown in Figure 1.

## 2. Endophytic Fungal Diversity

The diversity of endophytic fungi includes distribution diversity, species diversity, and secondary metabolite diversity.

Endophytic fungi can penetrate plant tissues through wounds, root system fractures, or natural apertures [7,8], in turn, colonizing all or a portion of the tissue, including the fruits, leaves, and stems. Consequently, endophytic fungi are found in practically every tissue of plants, and they may even be isolated from nodules in the roots [13]. The primary growth sites for endophytic fungal hyphae are the intercellular gaps of tissues, where they are most prevalent in seeds, leaf sheaths, and leaves.

The occurrence of endophytic fungi in various plant species also reflects the diversity of their dispersion. In the meantime, endophytic fungi may thrive in a variety of settings, such as marshes, forests, grasslands, and even aquatic ecosystems, due to their high environmental adaptability [9]. Further exploration of the factors influencing the distribution of endophytic fungi reveals that their distribution is influenced not only by plant taxonomy but also by geographical location and environmental conditions. Additionally, factors such as the surrounding soil, climate, and even the presence of neighboring plants can significantly affect the diversity of endophytic fungi within specific plant species (Table 1).

Aspergillus, Trichoderma, Fusarium, Penicillium, Colletotrichum, and Chaetomium are typical families and species of endophytic fungus. At least 35 taxa, 3 phyla, and 7 classes of endophytic fungi from Qinling *Rohdea chinensis* (Baker) N were isolated and identified [37]. About 153 species of endophytic fungi were isolated from Selaginella [38], of which 34 strains belonged to 7 genera of Ascomycetes, including Alternaria, Fusarium, Trichoderma, Penicillium, Aspergillus, and Phomopsis, as well as Mucor. The intrinsic characteristics of the host plants, such as their variety, isolation tissue, geographic location, growth period, and collecting season, are connected to the isolation frequency of endophytic fungi [39].

The variety of endophytic fungi found in different plants as well as within the same plant reflects the diversity of endophytic fungal families and genera. It is indicated that the endophytic fungi of sedum plants are concentrated in the stem of plants with rich species diversity, and 56 genera (orders) of endophytic fungi have been reported to have been isolated and identified from sedum plants, among which *Fusarium* sp. accounted for 19% of the total isolates, *Lasiodiplodia* sp. accounted for 17.26%, *Colletotrichum* sp. accounted for 4.68%, and *Trichoderma* sp. accounted for 3.24% [40].

**Figure 2:**
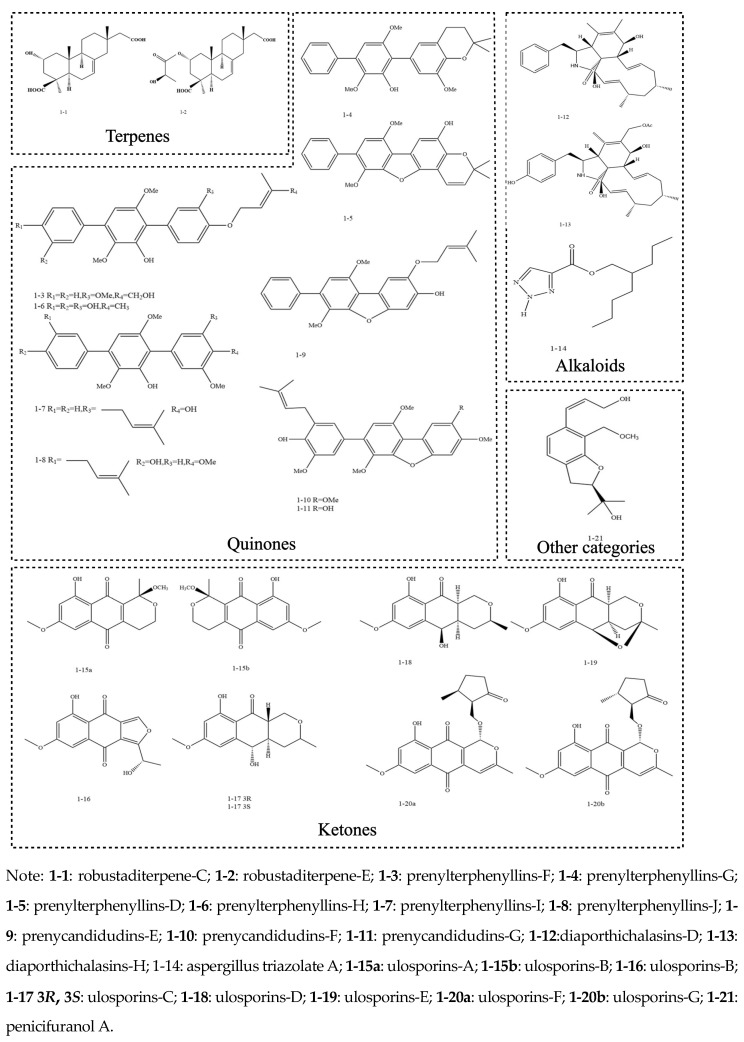
Main classification of secondary metabolites of endophytic fungi (adapted from [41,42,43,44,45,46,47]).

There are many different kinds of endophytic fungus that grow on plants. These endophytic fungi also create a wide variety of secondary metabolites, the most common of which are terpenoids (including sesquiterpenes and diterpenes), quinones, alkaloids, ketones, steroids, and so on (Figure 2).

## 3. Functional Diversity of Secondary Metabolites

### 3.1. Antimicrobial Effect

Given the declining efficacy of traditional antibiotics, endophytic fungi’s bioactivity could potentially develop into a novel class of antibacterial agent [48]. The antibacterial characteristics of certain endophytic fungi’s secondary metabolites are directly linked to the terpenoids and alkaloids. By competing with pathogens for living space, preventing the synthesis of bacterial cell membranes, obstructing essential growth processes [49], and breaking down bacterial cell walls, they prevent bacterial growth and reproduction [10].

Six endophytic fungal strains were isolated from *Hedera nepalensis* var. *sinensis* (Tobl.) Rehd [50]. The secondary metabolites’ dichloromethane/methanol extracts showed strong antibacterial activity against a range of bacterial strains. Eleusine indica’s endophytic fungi showed strong antagonistic activity against bacterial and fungal strains that were harmful [51]. Endophytic fungi isolated from Phyllanthus emblica fruits were discovered to have antibacterial activity [18]. Two endophytic fungi isolated from *Paris polyphylla* demonstrated significant antibacterial activity [52]. These endophytic fungi with antibacterial activity are new resources for the development of new antimicrobial drugs.

### 3.2. Anti-Tumor Effect

Endophytic fungal secondary metabolites demonstrate multiple modes of anti-tumor activity, such as decreasing tumor cell growth, causing apoptosis, lowering angiogenesis, regulating immunological responses, and preventing metastasis. Consequently, they can serve as a significant resource for novel anti-tumor active compounds.

It is indicated that abnormal expression levels of CDC25A can lead to cell cycle disruption, compromising DNA integrity and, as a result, the proliferation of malignant tumor cells [53]. Furthermore, CDC25B, a protein essential for cellular recovery, when over expressed, can facilitate tumor proliferation and malignant cell transformation. Consequently, CDC25A/CDC25B has emerged as a prominent target for the investigation and therapy of malignant neoplasms [54]. In recent years, PD-1/PD-L1 has emerged as a significant target for evaluating anti-tumor efficacy. Inhibiting the PD-1/PD-L1 relationship can stimulate the immune system to target tumor cells [55]. Endophytic fungal strains from Panax notoginseng exhibit CDC25A/CDC25B enzyme inhibitory activity and significant inhibition of PD-1/PD-L1 binding.

A metabolite from the WYJ-E14 strain of the fungus *Curcuma wenyujin* Y. H., which markedly inhibits the proliferation of melanoma and glioma cells, was identified [56]. The extracts from a specific endophytic fungus derived from lemon balm demonstrate cytotoxic effects on one or more human cancer cell lines [57]. Six compounds from the endophytic fungus *Fusarium oxysporum* LHS-P1-3 found in peanut roots, including beauvericin, enniatin, enniatin MK1688, and enniatin H, which demonstrate anticancer properties. It demonstrated that vinca alkaloids exhibit significant anti-tumor efficacy. Three endophytic fungi from the stems and tissues of periwinkle capable of synthesizing vincristine and vinblastine were isolated and identified, thus partially addressing the problem of resource scarcity [58].

### 3.3. Resistance to Insects

Endophytic fungus serve as eco-friendly pesticides by infiltrating insect bodies, multiplying extensively, interrupting metabolic processes, generating poisons, and finally resulting in their death. Concurrently, endophytic fungi can synthesize poisonous compounds, including alkaloids, that inhibit insect feeding. Endophytic fungus in plants may compete with insects for nutrients and influence reproductive processes, such as oviposition, thus contributing to pest resistance and management. Consequently, endophytic fungi possess significant potential for advancement in agriculture. The endophytic fungi of traditional insecticidal plants were investigated, discovering that their metabolites exhibit notable insecticidal properties. The endophytic fungi extracted from the root sections of cabbage possess insect-resistant characteristics and can enhance plant growth [59]. Endophytic fungi from Solanaceae plants harboring *Trichoderma asperellum* M2RT4, *Beauveria bassiana* ICIPE 706, and *Hypocrea lixii* F3ST1 exhibit insect-resistance properties [60]. These endophytic fungi have important value in the field of biological control.

### 3.4. Antioxidant Effect

Oxidative stress is vital in the occurrence and development of numerous illnesses. It is essential to develop more resources for the utilization of antioxidants in response to the situation. The secondary metabolites of endophytic fungi in plants comprise diverse bioactive compounds with antioxidant properties, potentially serving as novel antioxidant resources [61]. Endophytic fungi produce polyphenols, flavonoids, terpenes, and other compounds having antioxidant properties, which can substantially neutralize free radicals and mitigate oxidative damage [62]. These secondary metabolites help mitigate chronic diseases and prolong longevity by inhibiting cellular damage induced by free radicals and diminishing inflammation [63].

Upon detecting infections, plants promptly activate their oxidative systems, generating reactive oxygen species (ROS) as signals to initiate defense responses against the pathogens [64]. Endophytic fungi release enzymes that neutralize ROS during their interaction with plant hosts to mitigate stress and modulate immunological response. The GPI-anchored protein Ecm33-like, released by T. virens during colonization, can also safeguard hyphae from oxidative damage [65]. The existence of antioxidant compounds—flavonoids—in the metabolites of endophytic fungi derived from Bignoniaceae family plants was confirmed [66]. The endophytic fungi of stone algae and kelp had certain scavenging properties against DPPH radicals, hydroxyl radicals, and superoxide anions, with most demonstrating good antioxidant activity. Therefore, endophytic fungi can be considered a good resource for antioxidant-active compounds [67].

### 3.5. Anti-Inflammatory Effect

Chronic inflammation, as a possible catalyst for cardiovascular and autoimmune disorders, underscores the necessity for the development and investigation of novel anti-inflammatory agents. Metabolites from endophytic fungi, including phenolic and terpenoid chemicals, can modify inflammatory pathways, mitigate various effects contributing to chronic inflammation, and diminish disease risk [68]. The ethyl acetate extract of endophytic fungi derived from *Emilia sonchifolia* exhibits anti-inflammatory properties in vivo and in vitro [69]. Metabolites derived from endophytic fungi can inhibit nitric oxide production, demonstrate non-cytotoxic properties, and suppress the secretion of interleukin-6 (IL-6) and tumor necrosis factor-α (TNF-α) [70], thereby indirectly suggesting their anti-inflammatory activity.

Studies indicate that steroids has anti-inflammatory properties [71]; recently, the cholinergic anti-inflammatory pathway (CAP) has garnered significant attention [72]. Pharmacological tests were conducted on the steroid components in endophytic fungal metabolites to investigate the link between steroids and CAP [73]. The findings indicated that steroid metabolites elevated the expression of α7nAchR (nicotinic acetylcholine receptor) and suppressed the activation of its downstream signaling pathways, thus indirectly suggesting a potential link between steroids and CAP.

## 4. The Interaction Between Endophytic Fungi and Medicinal Plants

The origin of endophytic fungi is mostly elucidated by two theories: exogenous and endogenous. The endogenous idea posits that endophytic fungi derive from the chloroplasts and mitochondria of plants, sharing genetic material with the host organism. The exogenous hypothesis suggests that endophytes infiltrate the host plant through various pathways, including surface contact, induced channels, or root injuries [74]. The transpiration of plants can facilitate the movement of spores or hyphae of endophytic fungus within the plant, leading to the colonization of plant tissues [75]. Throughout their evolution, endophytic fungi and their host plants have established a variety of associations, which encompass mutualistic, antagonistic, and neutral interactions. Certain endophytic fungi largely remain dormant within host tissues throughout the entire life cycle of the plant, a condition referred to as neutral interaction. Some endophytic fungi can stay inactive until the right environmental conditions arise, resulting in either mutualistic or antagonistic relationships [76].

## 5. Promotion of Plant Growth

**Figure 3:**
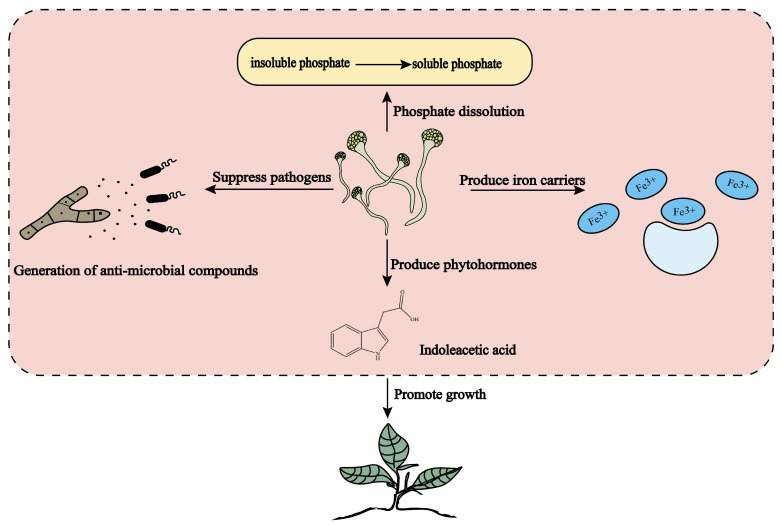
The direct mechanism of action of endophytic fungi (adapted from [77,78,79,80]).

A multitude of studies indicate that endophytic fungi can enhance plant growth both directly and indirectly through the production of diverse bioactive compounds. These include plant hormones like indole acetic acid and gibberellins, hydrolytic enzymes, iron carriers, phosphate solubilization, and antibacterial properties, which collectively contribute to the promotion of plant cell growth (Figure 3). Additionally, certain endophytic fungi possess the capability to fix nitrogen and solubilize minerals, which can enhance plant growth metrics and contribute significantly during the stages of plant growth and development.

### 5.1. Phytohormone Secretion

Auxin and ethylene are essential hormones in the symbiotic relationship between endophytic fungi and plants. The auxin synthesized by endophytic fungi can influence the development of plant roots [80]. Studies demonstrate that endophytic fungi can improve plant physiological performance by modulating hormone levels [81], therefore fostering vigorous growth of endophytes within the host and establishing a symbiotic connection [82]. IAA (Indole-3-acetic acid) is the predominant growth hormone in plants, facilitating growth; gibberellins (GAs), as a significant plant hormone, function throughout the entire plant lifecycle, promoting growth, germination, flowering, and fruiting, thereby enhancing fruit development and increasing yield.

The results indicated that seven endophytic fungi from the Melaleuca species were identified, which exhibited differing concentrations of IAA [83]. Subsequent study indicated that these endophytic fungi significantly enhance plant development. It has been observed that the endophytes associated with Antarctic pearl grass may influence the concentrations of jasmonic acid (JA), abscisic acid (ABA), salicylic acid (SA), and indole-3-acetic acid (IAA) in the early stem tissues of plants subjected to elevated UV-B radiation. This modulation appears to enhance plant productivity [82]. It was discovered that five strains of endophytic fungi from *Eleutherococcus senticosus* markedly enhanced seed germination. During stratification, the levels of plant growth regulators, including gibberellins (GAs), indole-3-acetic acid (IAA), indole butyric acid (IBA), and salicylic acid (SA), increased to varying extents, whereas the concentration of abscisic acid (ABA) exhibited a significant decline [84]. Furthermore, endophytic fungi has the capability to digest IAA, hence mitigating the inhibitory impact of elevated IAA concentrations on plant growth [85].

The plant hormone ethylene is synthesized by plants in reaction to stress in unfavorable conditions, and its elevated levels suppress plant development [86]. Certain endophytic fungi can synthesize ACC (1-aminocyclopropane-1-carboxylic acid) deaminase, which catalyzes the decomposition of ACC, the immediate precursor of ethylene production, into α-ketobutyrate and ammonia, consequently diminishing ethylene levels in plants and enhancing plant development [87]. Recent studies have focused on the activity of ACC deaminase, particularly in the context of endophytic fungi associated with plants, rhizosphere endophytic fungi, and strains that promote plant growth [88,89]. Endophytes belonging to the *Bacillus genus* have the capability to enhance plant growth through the production of ACC deaminase. This enzyme functions by inhibiting ethylene production, thereby reducing the concentration of ethylene within the plant.

### 5.2. Generation of Iron Carriers

Under conditions of soil pH, the concentration of soluble free iron ions is low, making it challenging for plants to absorb and utilize these ions effectively. Iron carriers represent a significant category of bioactive substances that possess the ability to efficiently and specifically chelate Fe^3+^. This process transforms Fe^3+^ into a form that is accessible for plant utilization, consequently facilitating enhanced plant growth. This could be a primary mechanism through which plants exhibit resistance to stress [78].

Endophytic bacteria are capable of synthesizing a variety of iron carriers, which include carboxylates, catecholates, phenolates, and hydroxamates. Iron carriers perform several functions, notably inhibiting plant pathogens by limiting their iron absorption, decreasing the toxicity of heavy metals present in the soil, and promoting the development of systemic resistance (ISR) [90,91]. Endophytic fungi that produce siderophores were identified, which enhance plant growth [92]. It was demonstrated that endophytic fungi that produce iron carriers can improve the growth indicators of spinach [93]. In total, 33 endophytic fungi from *Arnebia euchroma* were isolated, with 76.34% exhibiting the ability to produce siderophores and enhance plant growth [94].

### 5.3. Nitrogen Fixation

Nitrogen, an essential element for plant growth, is predominantly present in the atmosphere and is not directly usable by plants. Endophytic fungi possessing nitrogen-fixing capabilities are integral to plant health and development. Endophytic fungi possess the ability to convert atmospheric dinitrogen into trivalent nitrogen via their metabolic processes and nitrogenase, thereby supplying essential nitrogen for plant growth and development. Biological nitrogen fixation methods are categorized into three types: free-living nitrogen fixation, symbiotic nitrogen fixation, and associative symbiotic nitrogen fixation. Plant-root-associated bacteria (PGPR) with nitrogen-fixing capabilities include genera such as Azotobacter, Rhizobium, Azospirillum, Pseudomonas, and Klebsiella, among others. Rhizobium and Frankia are classified as obligate endophytic nitrogen-fixing bacteria, primarily associated with leguminous host plants [95].

In contrast, Pseudomonas is categorized as a facultative endophytic nitrogen-fixing bacterium. It was investigated that the impact of inoculating the endophytic fungus *Pseudomonas stutzeri* A15 on the growth of rice [96]. The research findings demonstrated that rice inoculated with Pseudomonas exhibited superior nitrogen-fixing capabilities compared to those treated with chemical nitrogen fertilizers, additionally enhancing rice growth. It was demonstrated that nitrogen-fixing Spirulina and Klebsiella, isolated from tobacco, promote the growth of the host plant [97]. A study to examine the potential of nitrogen-fixing bacteria, specifically Rhizobium and Bacillus, in enhancing the accumulation of active components in plants [98]. The roots of Astragalus were inoculated with a mixed inoculum of Rhizobium and Bacillus, followed by a metabolomics study on the metabolites present in the Astragalus roots. The findings demonstrate that applying Bacillus thuringiensis to the root tissue of root nodules enhances the production of growth-promoting plant hormones. This indirectly implies that both root nodules and Bacillus thuringiensis contribute to plant growth promotion and nitrogen fixation.

## 6. Solution of Phosphorus

Phosphorus is a crucial element for plant growth and development, serving as a key component in the synthesis of essential compounds and enhancing plants’ resistance to stress [99,100]. Approximately 95% of the phosphorus present in the soil exists in an inactive form, rendering it unavailable for direct absorption and utilization by plants.

Endophytic fungi mobilize inactive phosphorus in the soil via their metabolic byproducts, transforming it into a bioavailable form for plant uptake. Research shows that endophytic fungi solubilize phosphorus by secreting acidic substances, including organic acids and H^+^ ions. Phosphatases facilitate the release of phosphorus through the degradation of substrates. The respiration of inorganic salts and the assimilation of NH_4_^+^ by phosphorus-solubilizing endophytic fungi result in the release of H^+^, which causes a decrease in pH. The reduction in pH facilitates the binding of H^+^ ions with elements like iron, aluminum, and calcium, which in turn solubilizes certain insoluble phosphorus compounds in the soil.

The *Bacillus* genus represents the most efficient endophytes for the solubilization of phosphorus, which were extracted and screened plant-growth-promoting rhizobacteria (PGPR) from cotton roots that solubilize phosphorus and enhance cotton growth [79], identified as Bacillus. Research indicates that *Bacillus subtilis* exhibits significant phosphate solubilization capacity and can augment the synthesis of indole-3-acetic acid (IAA) and organic acids, hence improving plant resilience. The growth index of wheat with the integration of phosphates and *Bacillus subtilis* were markedly enhanced [101]. The endophytic fungus *Bacillus altitudinis* GQYP101, extracted from the roots of goji berries, have the ability to solubilize phosphorus and enhance the growth and development of corn. The examination of whole-genome sequencing of its growth-promoting processes indicated that this endophytic fungus is linked to phosphate solubilization, alkaline phosphatase activity, trendization, and motility [102].

## 7. Enhancement of Plant Resistance

**Figure 4:**
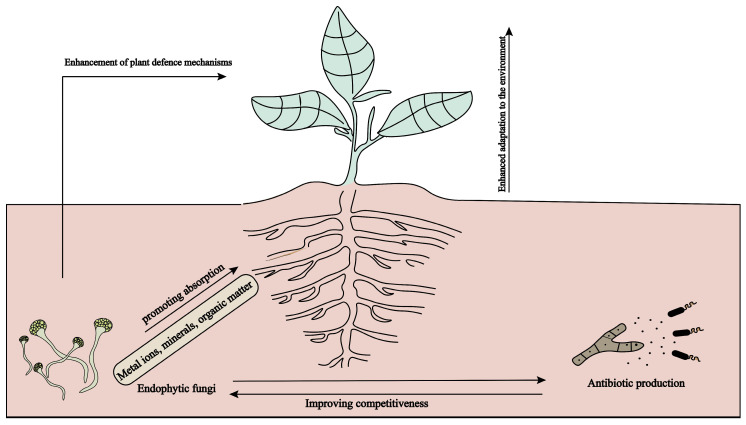
The mechanism of endophytic fungi enhancing plant stress tolerance.

Plants withstand detrimental environmental challenges throughout their development, encompassing biotic and abiotic stressors. Biotic stress mostly pertains to harm inflicted on plants by diseases, insects, and other creatures. Abiotic stress predominantly arises from drought, salinity, alkalinity, and heavy metals, resulting in nutritional depletion and metabolic dysfunction in host plants. Endophytic fungus can augment the resilience of host plants to biotic and abiotic conditions, particularly excelling in drought, salinity, alkalinity, and pest resistance [11], hence enhancing the survival capacity of medicinal plants. The ways in which endophytic fungi improve the stress resistance of medicinal plants can be classified into three categories (Figure 4): (1) through the facilitation of the host plant’s uptake of metal ions, minerals, and organic compounds, which in turn boosts the host plant’s resilience to environmental stress; (2) by synthesizing antibiotics or other secondary metabolites to bolster their own competitive edge; (3) by triggering or amplifying the host plant’s defense responses to fend off pathogen attacks.

The potential mechanisms through which the endophytic fungus *Seredipita indica* reacts to abiotic stress by regulating nutrient acquisition methods, osmotic adjustment, plant hormone levels, antioxidant enzyme defense systems, and functional genes [103].

The endophytic fungus *Penicillium ruqueforti* Thom., which is capable of producing IAA, can improve the resilience of wheat plants cultivated in soil abundant in heavy metals (nickel, cadmium, copper, zinc, and lead) against heavy metal contamination [92]. Additionally, wheat seedlings that are inoculated with endophytic fungi exhibit enhanced growth, increased nutrient absorption, and reduced levels of heavy metals in their stems and roots when irrigated with wastewater. Conversely, when exposed to heavy metal stress, the growth of uninoculated wheat plants is impeded, exhibiting indications of chlorophyll deficiency. The findings suggest that *Penicillium roqueforti* Thom. inoculation can foster a symbiotic relationship with host plants, supporting host crops in soils heavily contaminated with metals.

## 8. Promotion of the Synthesis and Accumulation of Metabolites in Plants

Endophytic fungi, upon invading medicinal plants, enhance the synthesis and accumulation of secondary metabolites in these plants. During the co-evolution of endophytic fungi and medicinal plants, endophytic fungal elicitors function as specific chemical signals that selectively and rapidly induce the expression of particular genes involved in the metabolic processes of medicinal plants. The activation of specific secondary metabolic pathways regulates the biosynthesis of metabolites in plants. This significantly enhances the synthesis and accumulation of active components. The endophytic fungi that produce IAA, glycosides, and cellulase were co-cultured with *Bletilla striata* (Thunb.) and were observed an increase in the total phenolic content in the medicinal parts of *Bletilla striata* (Thunb.) [12]. This finding suggests that these endophytic fungi enhance the synthesis and accumulation of metabolites within the plant.

Conversely, endophytic fungi co-evolve with plants within the plant body, resulting in gene recombination or the development of analogous signaling pathways in the endophytic fungi as those present in the host plants. This leads to the identification of secondary metabolites from endophytic fungi that are identical to those of their host plants, effectively addressing the issue of limited precious resources [104]. In 1993, Stierle et al. [105] identified endophytic fungi capable of producing paclitaxel, similar to the host plant Taxus.

## 9. Application of Omics Techniques in the Study Between Endophytic Fungi and Plants

Metagenomics, transcriptomics, proteomics, and metabolomics, as cutting-edge technologies for studying the interaction between medicinal plants and endophytic fungi, can be used to investigate the molecular mechanisms of the interaction. At the same time, each genomics technology has its advantages and limitations (Table 2). Metagenomics allows for microbial community profiling but lacks in-depth profiling of mechanisms. The use of transcriptomics and metatranscriptomics allows access to gene expression information but does not account for the entire genome or silenced genes. Proteomics provides direct insight into molecular mechanisms through protein expression, but may miss proteins of lower abundance. Metabolomics, on the other hand, offers functional data on metabolic pathways, but its data analysis is more complex. By combining these techniques, it is possible to provide a holistic view of plant–microbe interactions and reveal the complex mechanisms by which endophytic fungi affect the growth, health, and resistance of medicinal plants.

### 9.1. Metagenomics

Metagenomics offers sequence data regarding the microbial constituents of diverse biological communities [107]. The inability to culture certain endophytic fungi notably impacts the understanding of the interaction mechanisms between these fungi and plants [108]. This approach entails the extraction of DNA from the complete population for the analysis of its genetic composition. Niem et al. [109] employed Illumina sequencing to characterize the endomicrobiome linked to grapevine trunks and performed metagenomic analyses of endophytic bacterial and fungal communities independently. The data indicated differences in the diversity of bacterial and endophytic fungal communities in grapevines exhibiting trunk disease symptoms compared to those without, suggesting potential interactions between endophytic fungi and pathogens within the trunks of grapevines. Wijekoon et al. [110] employed ITS metagenomics to examine the diversity of endophytic fungal communities in three grape varieties, isolating and identifying endophytic fungi with bioactive and pathogenic characteristics. The advent of high-throughput sequencing (HTS) technologies has significantly enhanced our research and comprehension of microorganisms associated with plants. Analyzing sequence information of microorganisms within ecological communities and acquiring extensive data can improve our comprehension of the molecular mechanisms underlying plant–microbe interactions [111]. A model to study the principles of endophytic fungal interactions, utilizing endophytic fungi sourced from the stems and roots of Ephedra, was established [112]. A combined approach of metagenomics and metabolomics was employed to investigate the differences in the metabolic products of endophytic fungi in the stems and roots of *Ephedra*. The research findings demonstrate a significant correlation between endophytic fungi and various metabolites, highlighting the variability of secondary metabolites in specific tissues of endophytic fungi.

### 9.2. Transcriptomics and Macrotranscriptomics

Transcriptomics is a method for examining gene expression at the mRNA level, focusing on the comprehensive analysis of the complete set of mRNA present in cells or tissues under defined conditions. Transcriptomics examines the host transcriptome, whereas metatranscriptomics serves as an effective method for capturing gene expression within a community context.

Transcriptomics technology enables the examination patterns in gene expression of endophytes under stress conditions, facilitating a comprehensive understanding of the role of root exudates in the plant microbiome and elucidating the mechanisms of host immune regulation during biotic stress [111,113]. The endophytic fungus *Plectosphaerella cucumerina*, sourced from the plant *Rumex gmelinii* Turcz., enhances the growth of plant seedlings [114]. To investigate the mechanisms underlying this growth promotion, an in-depth study of its molecular mechanisms was conducted using transcriptomics. The findings indicated that the endophytic fungus significantly enhanced the expression of genes related to essential enzymes involved in amino acid metabolism and carbohydrate synthesis within the host plant, while also modulating the plant’s growth hormones. The endophytic fungus also stimulated the host plant to generate increased levels of stress-resistant compounds, thereby enhancing the growth and development of the host plant. A correlation analysis on paclitaxel biosynthesis utilizing endophytic fungi from Taxus, leading to the isolation of two endophytic fungal products, L7 and M14 [115]. Transcriptomics and non-targeted metabolomics studies revealed that L7 and M14 can modulate hormone signaling pathways, subsequently impacting the biosynthesis of paclitaxel.

### 9.3. Proteomics

Proteomics offers insights into the molecular interactions between plants and endophytic fungi by identifying and quantifying the proteins synthesized by endophytic fungi within host plant tissues. This clarifies the signaling pathways, metabolic changes, and biochemical processes involved in the adaptation of endophytic fungi to the plant growth environment [113].

For example, interactions between clumping arbuscular mycorrhizal fungi (AMF) and endophytic fungi can be analyzed through a combination of proteomics and transcriptomics [116]. It was shown that endophytic fungi significantly affect AMF growth and metabolism by regulating the pentose phosphate pathway and calcium signaling. Meanwhile, AMF and endophytic fungi showed similar metabolic adaptations in host plants, suggesting that their interactions are governed by multifactorial coordination. To unravel metabolite–protein networks at the host–fungus interface, ultra-performance liquid chromatography–mass spectrometry (UPLC-MS) allows for targeted quantitative analysis of key metabolites and functional linkage of these data to proteomic profiles. For example, UPLC-MS accurately detects endophytic fungal-induced accumulation of glucose-related metabolites in response to biotic stress in medicinal plants, thus confirming the up-regulation of glucose metabolizing enzymes revealed by proteomics [117]. Spatially, mass spectrometry imaging (MSI) techniques enable label-free visualization of protein–metabolite co-localization within plant tissues. In addition, nuclear magnetic resonance (NMR) can non-destructively resolve the dynamic turnover of metabolites in plant–fungal interaction systems.

### 9.4. Metabolomics

The examination of plant–microbe metabolites can function as biomarkers for investigating the responses of medicinal plants and endophytic fungi under stress situations.

UPLC, when coupled with tandem mass spectrometry (UPLC-ESI–MS/MS), significantly enhances chromatographic resolution and sensitivity, enabling the rapid separation and detection of low-abundance metabolites in complex plant–fungal systems. This approach is particularly valuable for untargeted metabolite profiling in symbiotic interactions, as it facilitates the discovery of stress-responsive compounds with distinct chemical characteristics, such as alkaloids or terpenoids produced during host–endophyte crosstalk [118]. Concurrently, focused approaches are frequently employed in metabolomics research. Tandem mass spectrometry is extensively utilized in targeted methodologies for the quantitative assessment of identified compounds, ensuring high specificity for validating candidate biomarkers. Analytical methods such as NMR, gas chromatography, liquid chromatography, and capillary electrophoresis may be employed singularly or in conjunction with other techniques. For instance, NMR spectroscopy provides non-destructive structural elucidation of metabolites, making it indispensable for characterizing volatile or unstable compounds in endophytic fungi–plant systems [119].

The spatial distribution of metabolites within the tissues of medicinal plants is essential to study the role of specific metabolites and their interaction with the host plant. MSI, including MALDI-MSI, allows the localization of metabolites to be observed at the cellular or tissue level. The technique desorbs and ionizes metabolites directly from the surface of the sample, eliminating the need for prior labeling and allowing for multiple compounds to be mapped simultaneously. For example, MALDI-MSI has been applied to resolve the heterogeneous chemical landscapes of Streptomyces endophytes within plant tissues, revealing niche-specific production of antimicrobial metabolites at fungal colonization sites [120]. Complementary to this, magnetic resonance imaging (such as MRI) non-invasively captures metabolic gradients in intact plant tissues by detecting nuclear spin signals, providing dynamic insights into metabolite transport and compartmentalization during symbiotic interactions. This multimodal integration of UPLC-MS, MSI, and NMR achieves a systematic understanding of the phyto-endophytic fungal metabolic exchanges in medicinal plants.

A comprehensive understanding of the spatial distribution of metabolites and their chemical composition in medicinal plants and endophytic fungi will enhance the knowledge to study the interactions between these entities. UPLC-ESI-MS/MS excels at high-throughput metabolite annotation, whereas MSI and NMR imaging address the key limitations of traditional metabolomics by preserving a stereoscopic image of their structure. However, challenges remain: MSI requires optimization of matrix application to avoid bias in medicinal plant-endophyte fungi samples, while NMR imaging is less sensitive compared to MS-based techniques. Strategically combining these techniques—such as using NMR for structural validation and UPLC-MS for quantitative analyses—can mitigate their respective shortcomings for the study of mechanisms of medicinal plant-endophytic fungal symbiotic interactions.

### 9.5. Final Remarks

Medicinal plants are generally used for medical and pharmaceutical purposes, and their endophytic fungi have the more opportunity to produce the same or similar medicinal products, such as antibacterial, anti-tumor, antioxidant, etc. Its mechanism of action may be related to the co-relationship between endophytic fungi and medicinal plants [121]. Endophytes can not only participate in the synthesis of secondary metabolism of host plants but also independently complete the synthesis of secondary metabolites. In the study of the interaction between endophytic fungi and host, the application of omics technology greatly improves the research efficiency. Recently, more and more researchers are using the combined techniques of omics, namely multi-omics, to solve scientific problems [122]. Through the combined use of genomics, transcriptomics, proteomics, metabolomics, and other technologies in part or in whole, and interactive verification, life phenomena can be comprehensively and systematically explained from various levels such as gene, transcription, expression, accumulation of secondary metabolites, and epigenetics, thus accelerating the research process in the field of endophyte–plant interaction [123]. In this paper, the research progress of endophytic fungi was comprehensively reviewed from the perspectives of diversity of endophytic fungi at different levels, the relationship between endophytic fungi and their hosts in medicinal plants, as well as new technologies and new methods. In addition, the application of new technologies, especially omics, in the field of endofungal research was also fully discussed.

## 10. Conclusions

The diversity of endophytic fungi, not only in terms of their distribution and species but also in terms of their secondary metabolites, provides greater opportunities for the future development of endophytic fungal research. Secondary metabolites of plant endophytic fungi exhibit a variety of beneficial effects, including antimicrobial, anti-tumor, anti-inflammatory, and antioxidant properties, making them promising therapeutic drug candidates and providing a new resource for the drug development and pharmaceutical fields. Meanwhile, some secondary metabolites of endophytic fungi have insecticidal effects and can be used to formulate novel biopesticides. Research on the mechanism of interaction between endophytic fungi and medicinal plants has shown that some endophytic fungi improve the growth indexes of medicinal plants by secreting hormones to solubilize scales, fixing nitrogen and providing iron carriers to promote plant growth, and that endophytic fungi can improve the resistance of medicinal plants and contribute to the accumulation of secondary metabolites, so the endophytic fungal resources can be used as a bio-fertilizer and bioremediation agent to enhance the health of plants, increase the yield of crops, and improve the quality of the soil quality development resources. Endophytic fungi can parasitize medicinal plants without compromising their health, thus establishing a mutually beneficial symbiotic relationship. In this interaction, endophytic fungi can directly or indirectly promote the growth of medicinal plants. Maximizing the ecological role of endophytic fungi can reduce dependence on pesticides and fertilizers and promote sustainable agriculture.

The combined application of omics techniques promises to advance the understanding of the intricate interactions between medicinal plants and their endophytic fungi. By integrating metagenomics, transcriptomics, proteomics, and metabolomics, these interactions can be explored at the molecular level to reveal the mechanisms underlying plant health, growth, and resistance. As these technologies continue to evolve, they will provide valuable insights into how plants and their endophytic fungal communities can be utilized for sustainable agricultural practices and the discovery of novel bioactive compounds.

## Figures and Tables

**Table 1:** Secondary metabolites of endophytic fungi and their host plants.

Endophytic Fungi	Plant Host	Bioactivity	Secondary Metabolites/Enzymes	Reference
*Bionectria* sp.	*Huperzia serrata* (Thunb. ex Murray) Trevis.	Antimicrobial	Bionectin D	[14]
*Hormonema* sp.	*Juniperus communis*	Antimicrobial	cyclic dipeptides	[15]
*Chaetomium* sp.	*Astragalus chinensis*	Antimicrobial	differanisole A, 2,6-dichloro-4-propylphenol and 4,5-dimethylresorcinol	[16]
*Aspergillus ochraceus*	*Lagopsis supina*	Antimicrobial	2-methoxy-6-methyl-1,4-benzoquinone	[17]
*Aspergillus fumigatus*	*Dillenia indica*	Antimicrobial	2-isopropyl-5-methyl-1-heptanol, dodecane, 1-fluoro-pentanoic acid, 2-ethylhexyl ester, 1-octanol, 2-butyl-1-dodecanol	[18]
*Colletotrichum* sp.	*Andrographis paniculata* (Burm. f.) Nees	Antimicrobial	diterpene lactones	[19]
*Cochliobolus hawaiiensis*	*Millingtonia hortensis* L. f.	Anti-tumor	taxol	[20]
*Alternaria* Nees	*Passiflora incarnata* L.	Anti-tumor	chrysin	[21]
*Alternaria alstroemeria*	*Artemisia artemisia*	Anti-tumor	AaLaeA^OE26^	[22]
*Aspergillus niger*	*Ficus microcarpa* L. f.	Resistance to insects	*N*-[1-(4-methoxy-6-oxopyran-2-yl)-2-methylbutyl] acetamide	[23]
*Paecilomyces* sp.	*Schnella splendens*	Resistance to insects	Phomoxanthone A	[24]
*Aspergillus terreus*	*Catharanthus roseus*	Resistance to insects	phenolic	[25]
*Aspergillus versicolor*	*Cyclocarya paliurus* (Batal.) Iljinsk.	Antioxidant	exopolysaccharides	[26]
*Aspergillus flavus*	*Silybum marianum*	Antioxidant	kojic acid	[27]
*Aspergillus nidulans* var. *dentatus*	*Passiflora incarnata*	Antioxidant	tannins	[28]
*Lasiodiplodia theobromae*	*Cymbidium* sp.	Antioxidant	-	[29]
*Aspergillus fumigatus*	*Dillenia indica*	Antioxidant	2-isopropyl-5-methyl-1-heptanol, dodecane, 1-fluoro-pentanoic acid, 2-ethylhexyl ester, 1-octanol, 2-butyl-1-dodecanol	[18]
*Colletotrichum* sp.	*Andrographis paniculata* (Burm. f.) Nees	Antioxidant	diterpene lactones	[19]
*Lasiodiplodia* sp.	*Handroanthus impetiginosus*	Antioxidant	-	[30]
*Aspergillus versicolor*	*Juncus rigidus*	Antioxidant	Physcion	[31]
*Acremonium alternatum*	*Argemone mexicana*	Anti-inflammatory	aconitine	[32]
*Biscogniauxia petrensis*	*Dendrobium orchids*	Anti-inflammatory	sesquiterpenoids	[33]
*Stagonosporopsis oculihominis*	*Dendrobium huoshanense*	Anti-inflammatory	(*Z*)-1-hydroxy-4-(2-nitroethenyl)-benzene, *p*-hydroxybenzaldehyde and *p*-hydroxyphenylacetic acid	[34]
*Setosphaeria rostrata*	*Ipomoea pes-caprae*	Anti-inflammatory	ravenelin	[35]
*Aspergillus niger*	*Elaeocarpus floribundus* Blume	Anti-inflammatory	nafuredin	[36]
*Aspergillus versicolor*	*Juncus rigidus*	Anti-inflammatory	Physcion	[31]

**Table 2:** Comparative analysis of omics techniques. (adapted from [106]).

Omics	Advantages	Disadvantages	Advantages and Disadvantages of Related Technologies
Metagenomics	No need to culture microorganisms	The samples are susceptible to contamination	16S rRNA gene sequencing Advantages: Low-cost, simple data analysis; highly targeted; wide range of applications Disadvantages: Limited information; low sensitivity
	Comprehensiveness	High cost	
	High throughput	Complexity in analyzing results	Genome-wide association technology (GWAS) Advantages: Comprehensive; unbiased; high resolution; widely used Disadvantages: Only common variants can be detected; population-dependent; unclear functional mechanism; high sample and cost requirements
	Functional gene discovery	Limited species resolution	
Transcriptomics and Macrotranscriptomics	High sensitivities	Complexity of data analysis	scRNA-seq Advantages: High accuracy; high specificity; ability to conduct unbiased high-throughput studies with minimal sample starts Disadvantages: High sample quality required; only RNA with poly(A) can be analyzed
	Without prejudice	High sample handling requirements	RNA-Seq Advantages: High throughput; high accuracy; wide detection range; low cost Disadvantages: Inability to drive the heterogeneity of individual cell expression
	Comprehensiveness	Higher costs	Gene chip technology Advantages: Automation; anti-contamination; high throughput; miniaturization Disadvantages: Cannot be applied to species with incomplete whole genome sequencing and poor gene annotation information; low abundance of expressed genes is not easily accessible
	Wide range of applications	Technical discrepancy	Expressed Sequence Tagging Technology Advantages: PCR products are of the same size in both DNA and genomic templates; easy to separate individual genes from members of similar gene families with closely related coding sequences Disadvantages: High error rate; low abundance of expressed genes are not suitable for obtaining; ESTs are very short and do not give complete sequence expression
Proteomics	Dynamism	Higher costs	2-dimensional gel electrophoresis Advantages: High throughput; high resolution; good repeatability Disadvantages: Only soluble proteins can be analyzed; high workload; sample proteins required
	Comprehensiveness	Sample heterogeneity	ICAT Advantages: Separation is performed at the peptide level, solving the problem of proteolysis and allowing the identification and quantification of membrane proteins; peptides containing multiple cysteines can be identified and quantified; low abundance proteins can be directly measured and identified Disadvantages: ICAT has a molecular weight of approximately 500 Da, which affects the identification of small peptides; it is not possible to analyze proteins that do not contain Cys; it does not accurately reflect the true content of proteins in the cell
	Wide range of applications	Complexity of data analysis	iTRAQ Advantages: Accurate quantification; high sensitivity; diverse samples; low abundance of proteins, strong basic proteins Disadvantages: high cost
	High sensitivity	Technical complexity	Label-Free Quantitative Proteomics Advantages: Low cost; simple treatment process Disadvantages: Low process accuracy; dependence on mass spectrometry experimental reproduction
Metabolomics	Functional directness	Technically complex	GC-MS Advantages: High resolution; high sensitivity; enables structural identification of compounds; low cost Disadvantages: Inability to separate large molecules; inability to detect substances that cannot be gasified; time-consuming derivatisation process
	High sensitivity	Complexity of data analysis	LC-MS Advantages: High separation efficiency; fast analysis; can separate structurally similar compounds Disadvantages: Need to change columns as appropriate; limited number of metabolite species analyzed
	Wide range of applications	Limited dynamic range	NMR Advantages: Low sample volume; no sample pre-treatment required Disadvantages: Low sensitivity and resolution; high requirements for sample preparation; limited dynamic range
	Dynamism	High cost	CE-MS Advantages: Small sample size; wide metabolite coverage Disadvantages: Poor reproducibility of separation; narrow linear range for quantitative analysis

## Data Availability

No new data were created or analyzed in this study.
